# Supplementary material for: Effects of Doxorubicin Delivery by Nitrogen-Doped Graphene Quantum Dots on Cancer Cell Growth: Experimental Study and Mathematical Modeling
Source: Nanomaterials (Basel). 2021 Jan 8;11(1):140. doi: 10.3390/nano11010140 (PMC7827955; doi:10.3390/nano11010140)
Supplement: Supplementary file 1 [file nanomaterials-11-00140-s001.pdf]

nanomaterials

Effects of Doxorubicin Delivery by Nitrogen-Doped Graphene Quantum Dots on Cancer Cell Growth: Experimental Study and Mathematical Modeling

Madison Frieler [1], Christine Pho [2], Bong Han Lee [2], Hana Dobrovolny [2], Giridhar R. Akkaraju [1] and Anton V. Naumov [2,*]

[1] Department of Biology, Texas Christian University, Fort Worth, TX 76129, USA; madison.frieler@tcu.edu (M.F.); g.akkaraju@tcu.edu (G.R.A.)
[2] Department of Physics and Astronomy, Texas Christian University, Fort Worth, TX 76129, USA; christine.pho@tcu.edu (C.P.); bong.lee@tcu.edu (B.H.L.); h.dobrovolny@tcu.edu (H.D.); a.naumov@tcu.edu (A.V.N.)
* Correspondence: a.naumov@tcu.edu

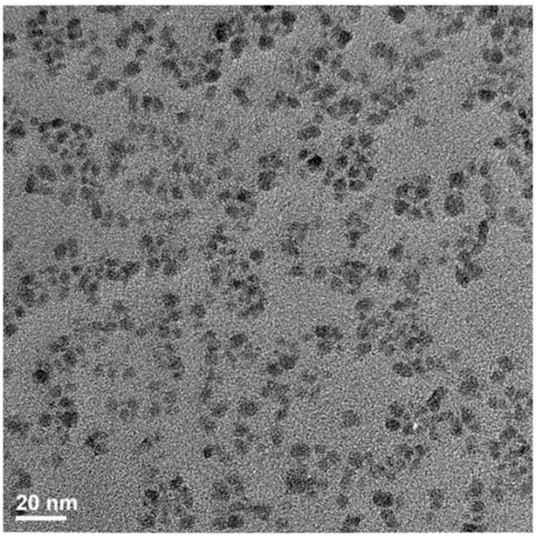

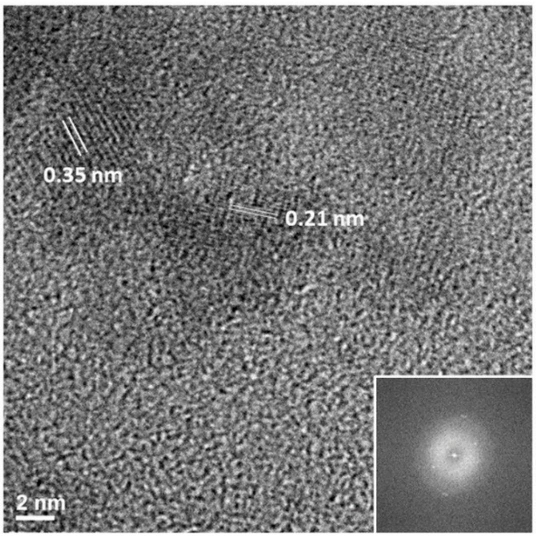

(a) (b)

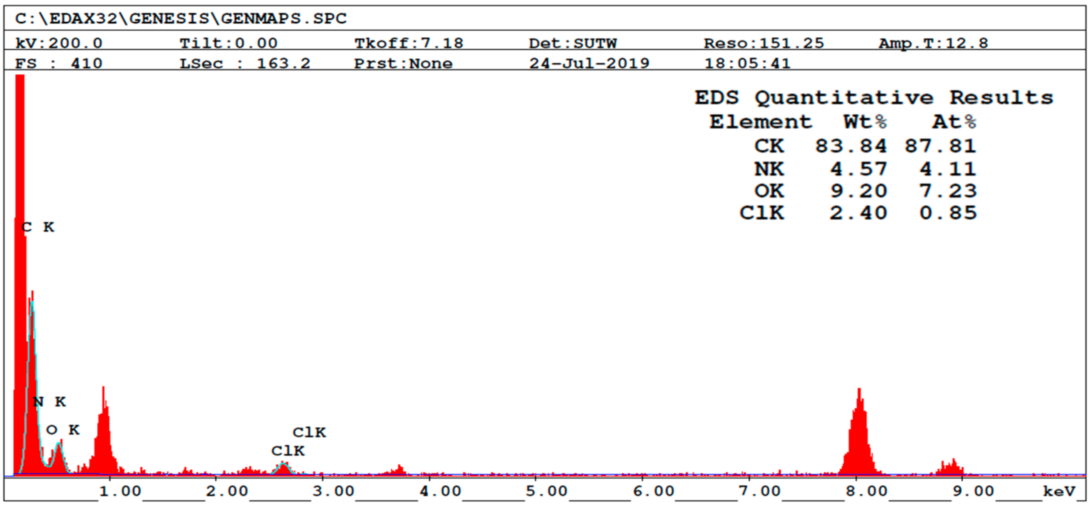

(c)

Figure S1. (**a**) TEM image of N-GQDs. (**b**) HRTEM image of N-GQDs. (**c**) EDX of N-GQDs.

For the structural characterization of N-GQDs, transmission electron microscope (TEM) and high resolution TEM (HRTEM) have been utilized. Based on the fast-Fourier-transform (FFT) (inset of Figure S1b), high crystalline structure of N-GQDs are noticeable. Lattice fringes of 0.21 and 0.35 nm spacings are determined that respectively correspond to the (100) in and (002) basal plane spacing of graphite.

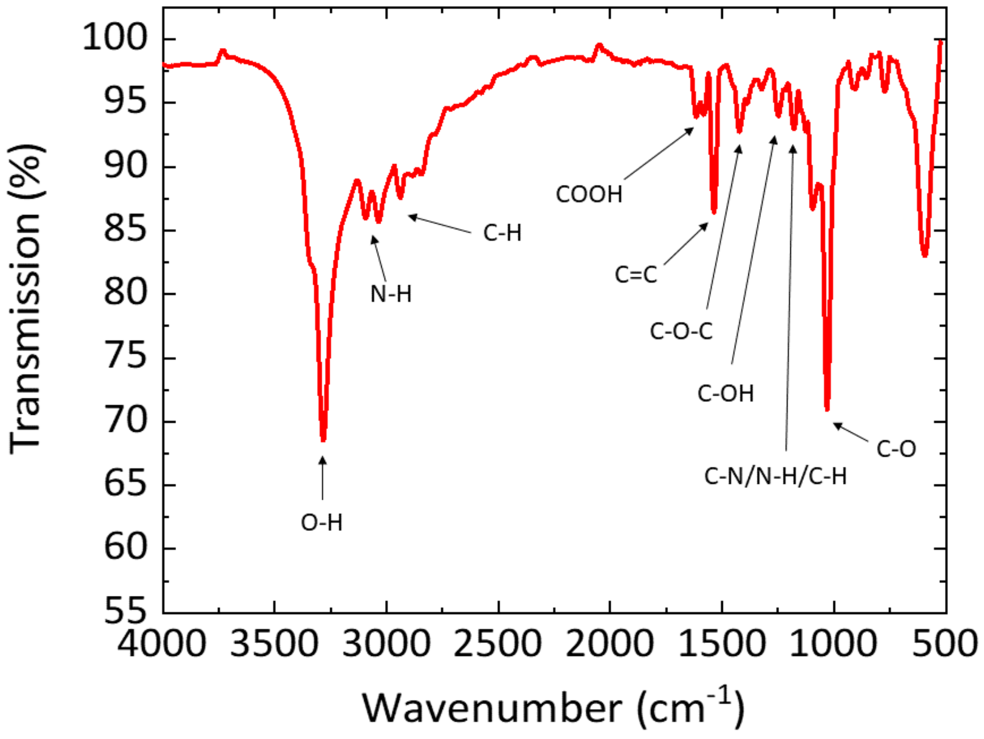

Figure S2. FTIR spectra of N-GQDs.

For the chemical composition of N-GQDs, we have determined the functional groups present in N-GQDs using the Fourier-transform infrared spectroscopy (FTIR) with an attenuated total reflectance (ATR) accessory; these N-GQDs have been freeze dried prior measurement. From the spectra, stretching vibrations are observed that correspond to the O-H, N-H, C-OH, C-N/N-H/C-H, and C-O groups at 3275, 3090, 1330, 1240, and 1021 cm^{-1}, respectively. Moreover, transition vibrations corresponding to C-H, C=O from COOH, C=C, and C-O-C groups at 2935, 1602, 1530, and 1412 cm^{-1}.

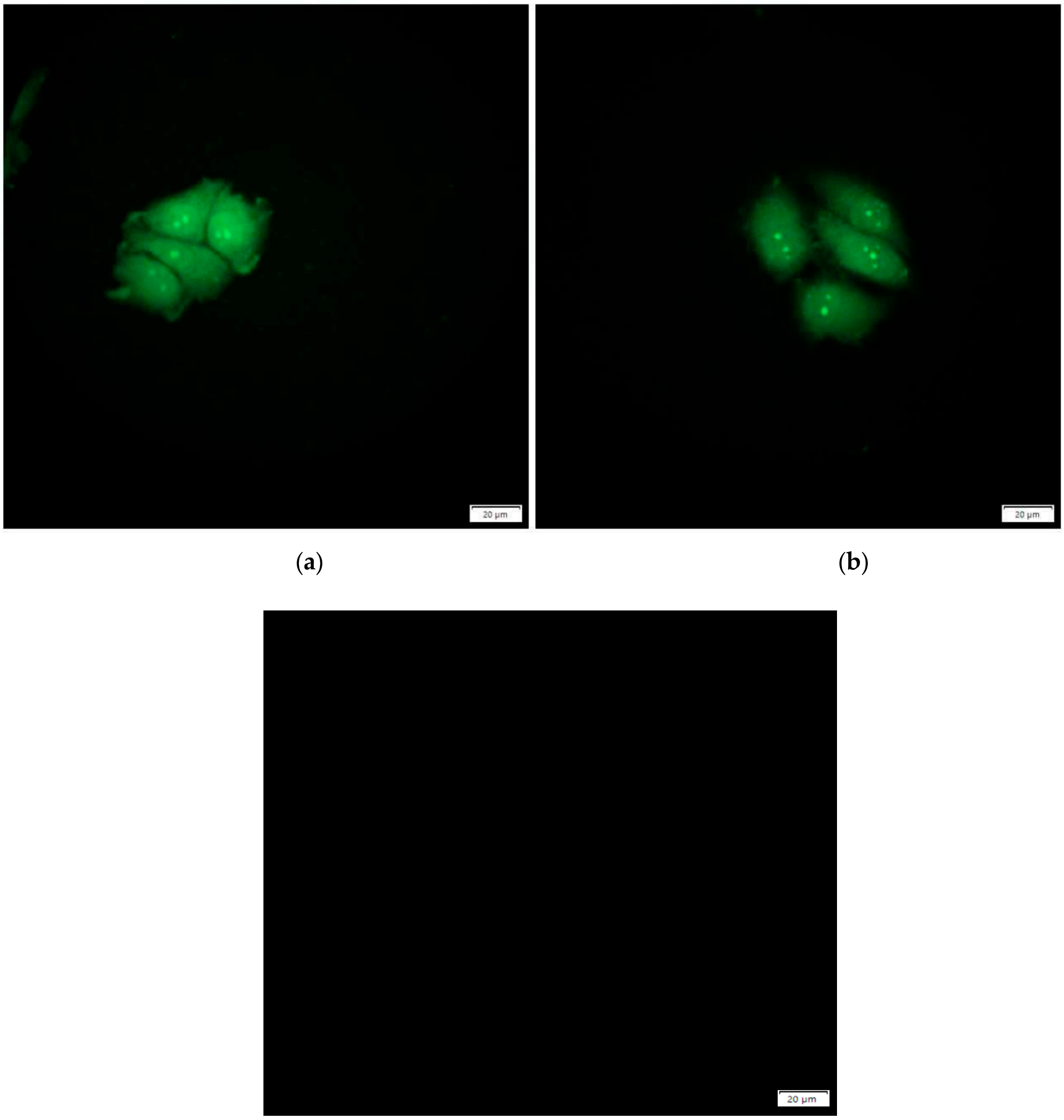

(**a**) (**b**)

(**c**)

Figure S3. (**a**,**b**) Confocal fluorescence images of N-GQD fluorescence originating from DOX- N-GQDs in MCF-7 cells at 3 h time point. (**c**) nontreatment control fluorescence.
